# Enhancement of Local Photovoltaic Current at Ferroelectric Domain Walls in BiFeO_3_

**DOI:** 10.1038/srep43070

**Published:** 2017-02-20

**Authors:** Ming-Min Yang, Akash Bhatnagar, Zheng-Dong Luo, Marin Alexe

**Affiliations:** 1 Department of Physics, University of Warwick, Coventry, CV4 7AL, United Kingdom; 2 Centre for Innovation Competence SiLi-Nano, Karl-Freiherr-von-Fritsch-Straße 3, D-06120 Halle (Saale), Germany

## Abstract

Domain walls, which are intrinsically two dimensional nano-objects exhibiting nontrivial electronic and magnetic behaviours, have been proven to play a crucial role in photovoltaic properties of ferroelectrics. Despite this recognition, the electronic properties of domain walls under illumination until now have been accessible only to macroscopic studies and their effects upon the conduction of photovoltaic current still remain elusive. The lack of understanding hinders the developing of nanoscale devices based on ferroelectric domain walls. Here, we directly characterize the local photovoltaic and photoconductive properties of 71° domain walls on BiFeO_3_ thin films with a nanoscale resolution. Local photovoltaic current, proven to be driven by the bulk photovoltaic effect, has been probed over the whole illuminated surface by using a specially designed photoelectric atomic force microscopy and found to be significantly enhanced at domain walls. Additionally, spatially resolved photoconductive current distribution reveals a higher density of excited carriers at domain walls in comparison with domains. Our measurements demonstrate that domain wall enhanced photovoltaic current originates from its high conduction rather than the internal electric field. This photoconduction facilitated local photovoltaic current is likely to be a universal property of topological defects in ferroelectric semiconductors.

Domain walls (DWs) in ferroelectrics have attracted increased attention in recent years owing to their unique physical properties, such as enhanced electronic conduction^1^,^2^,^3^,^4^, magnetoelectric coupling^5^,^6^, and the capability of the manipulation by external electrical, magnetic or strain field^7^. Additionally, DWs play a crucial role in the macroscopic properties of their host materials, such as polarization switching^8^, permittivity^9^ and pyroelectric properties^10^. In particular, they have emerged as key focal interests in the field of photo-ferroelectrics since the discovery of the anomalous photovoltaic effect (APV) in bismuth ferrite (BiFeO_3_, BFO) thin film^11^,^12^. It was firstly proposed that domain walls were the origin of above band gap open-circuit voltage (*V*_*OC*_) owing to the efficient separation of electrons and holes by the internal electric field within domain walls^11^,^12^. However, subsequent experiment revealed that the actual mechanism behind APV effect is the bulk photovoltaic effect (BPV) resulting from its non-centrosymmetric lattice structure^13^. In contrast to the known photovoltaic effect in semiconductors with p-n junctions, the photovoltaic (PV) current in the BPV effect arises from the asymmetric momentum distribution of the non-equilibrium carriers in the bulk and the *V*_*OC*_ is inversely proportional to the conductivity under illumination^14^. BPV effect in ferroelectric materials leads to an anomalously large *V*_*OC*_, i.e. well beyond the value of the forbidden bandgap, which itself can affect the indices of the refraction (photorefractive effect)^15^ and other physical properties^16^. However, domain walls would instead supress the value of *V*_*OC*_ owing to the enhanced conductivity^13^. Nevertheless, the performance of a ferroelectric solar cells is not only determined by the value of *V*_*OC*_ but also the magnitude of the PV current. The question that whether domain walls would facilitate or hinder the conduction of the BPV current, however, still remains unresolved, despite its importance for the photovoltaic performance based on ferroelectric materials.

In fact, it is still a challenge to distinguish the photo-response of domain walls in a ferroelectric device by the macroscopic studies due to their coexistence with domain matrix. In order to address this problem, it is crucial to characterize the local photo-response of ferroelectric materials with a nanoscale resolution, providing thus insight into electronic properties of each entities, i.e., domain and domain wall, in a ferroelectric PV device.

Here, we present a systematically study of the local photovoltaic properties of BFO thin films with a nanoscale resolution, which reveals an enhancement of PV current at domain walls. The studied BFO films consist of pure 71° domain walls and exhibit substantial BPV effect, i.e., above bandgap *V*_*OC*_ and light-polarization dependent PV current, at both macroscopic and nanoscale levels. Domain wall-enhanced local PV current is observed in two different sample geometries, i.e., DWs parallel and perpendicular to the grounded electrodes. Furthermore, using the ability to tune the BPV effect by varying the light polarization angle, spatially resolved photoconductive current is mapped which indicates a higher density of photo-excited carriers at domain walls compared to that of domains. The resultant enhancement of conduction of domain walls effectively facilitates the transport and collection of PV current originated from the BPV effect.

## Results

### Thin film fabrication

**Figure 1:**
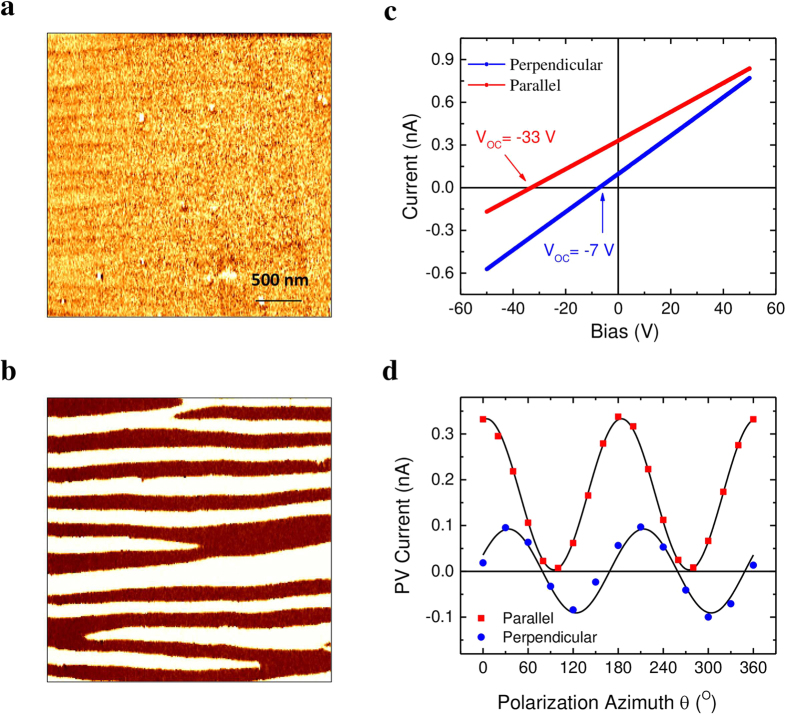
Domain pattern and macroscopic photovoltaic characterization. (**a**) Out-of-plane PFM phase signal indicating a uniform out-of-plane polarization direction and (**b)** in-plane PFM phase signal showing ±90 phase change for alternative polarization directions in each domains. (**c)** Macroscopic *I-V* characteristics under illumination from a 405 nm laser with in-plane electrodes aligned parallel to (red line) and perpendicular to (blue line) the 71° domain walls. (**d)** Variation of photovoltaic current as function of the angle between light polarization and the in-plane net ferroelectric polarization with in-plane electrodes running parallel (red square dots) and perpendicular (blue circle dots) to domain walls. The continuous black lines are the fitting with Equations 2 and 3 for parallel and perpendicular geometry, respectively.

Nominally 200–300 nm thick (001)-oriented BFO films were deposited epitaxially on bare (110)-oriented TbScO_3_ substrates by pulsed laser deposition technique. In order to get stripe domains comprising only 71° DWs, TbScO_3_ substrates were annealed in 1000 °C with O_2_ flow for 2 h before deposition^11^. Details on the deposition parameters and structure characterization are given in Methods and Supplementary Figure S1. Figure 1a and b show the out-of-plane and in-plane PFM phase images, respectively, indicating the well-defined 71° stripe domains with an averaged width of about 160 nm.

### Macroscopic characterization of PV effect

In order to confirm that the films under investigation show a typical behaviour, we first characterized the macroscopic photovoltaic properties of the BFO film by employing in-plane electrodes in two different geometries: electrodes parallel and perpendicular to the DWs, respectively (see Supplementary Fig. S2). As illustrated in Fig. 1c, the BFO film exhibits substantial APV effect in both geometries with above bandgap (E_g_ ~ 2.7 eV) open circuit voltage (*V*_*OC*_) and large short-circuit current (*I*_*SC*_). Specifically, *V*_*OC*_ and *I*_*SC*_ reach −33 V and 330 pA in the parallel geometry, −7 V and 100 pA in the perpendicular geometry, respectively. To get further insight into the APV effect of BFO film, the PV current was measured by varying laser polarization angle with respect to the orientation of BFO film while keeping the illumination intensity constant. Through rotating the light polarization using a half wavelength (λ/2) plate, the values of *I*_*SC*_ were recorded simultaneously with the azimuthal angle *θ* between light polarization and the net in-plane ferroelectric polarization. Figure 1d shows the variation of *I*_*SC*_ along with laser polarization azimuth in the two different electrode geometries. In the parallel geometry, the *I*_*SC*_ reaches maximum when laser polarization is perpendicular to the DWs while decrease to almost zero as the light polarization runs parallel to the DWs. Additionally, the sign of the *I*_*SC*_, viz. the current direction, can be tailored along with its magnitude by the incident light polarization in the perpendicular geometry. This peculiar light polarization dependent PV current clearly differentiates the APV effect of BFO film from the conventional photovoltaic effect as in the classic p-n junction. In a conventional PV effect in which separation of the non-equilibrium photo-generated carriers is based on a gradient of the chemical potential, the PV current does not depend on light polarization. This dependence is a strong indication that behind mechanism of the APV effect is the bulk photovoltaic effect (BPV). The PV current density *J*_*i*_ in this case can be expressed in the form given by^17^


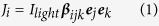


where ***β***_*ijk*_ is a third rank BPV tensor and *I*_*light*_ the light intensity, ***e***_*j*_ and ***e***_*k*_ the projection of the electrical field of the light onto the BFO film. Concerning the 71° domain configuration of the studied BFO film, the response of BPV effect in parallel and perpendicular geometries can be expressed by the following equations respectively^13^,^18^:


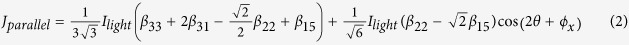



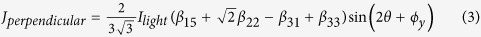


where *J*_*parallel*_ and *J*_*perpendicular*_ are the current density in parallel and perpendicular geometry, respectively; *β*_*ij*_ the BPV tensor elements expressed in the matrix notation (see Supplementary Table S1); *ϕ*_x_ and *ϕ*_y_ the phase shift to compensate experimental effects, for instance, misalignment between the in-plane net ferroelectric polarization and light polarization when *θ* = 0°. The above equations fit very well the experimental data (see Fig. 1d), confirming that the mechanism of APV effect in BFO film is the BPV effect.

### Nanoscale characterization of local PV effect

**Figure 2:**
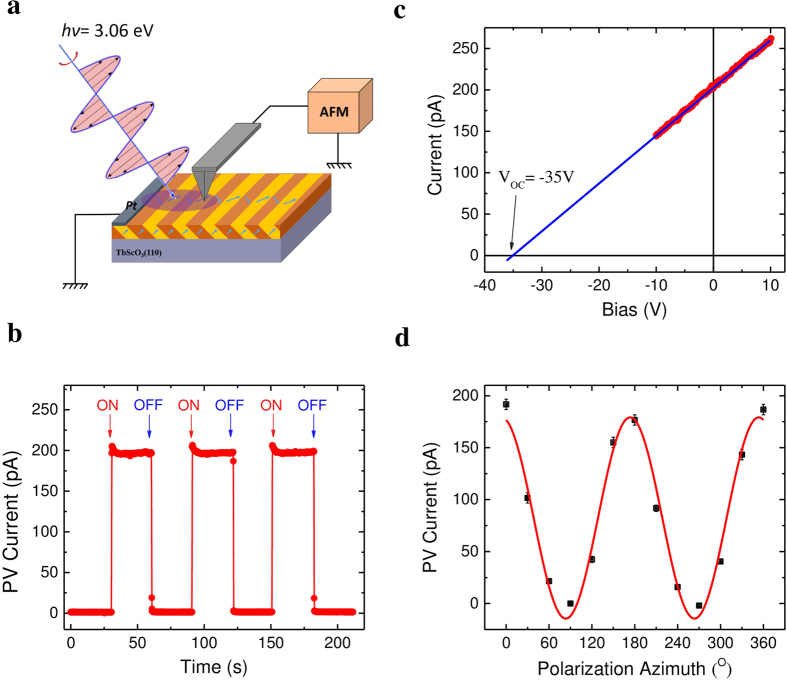
Local photovoltaic characterization. (**a**) Schematic showing the local measurement conducted by photoelectric atomic force microscopy (PhAFM) under illumination. (**b**) Photovoltaic current probed by PhAFM tip. (**c)** Local *I-V* characteristics measured through the PhAFM tip. The linear exploration gives -35V as open circuit voltage. (**d)** Photovoltaic current collected by PhAFM tip as a function of light polarization. The continuous red line is the fitting with Equation 2.

Our main goal is to explore to the local photoelectric properties with a nanoscale resolution in order to understand the role of the domain walls in the PV effect, especially the transport of PV current. For this purpose, we have used a home-build photoelectric atomic force microscopy (PhAFM). As depicted in Supplementary Fig. S3, PhAFM consists of an AFM-based system modified by a custom current amplifier/filter system and an optical system. The latter allows illumination of the sample surface with tunable light polarizations by employing a λ/2 plate. As schematically shown in Fig. 2a, a platinum stripe electrode was deposited on the BFO surface to close the current circuit with the AFM tip. Note here that the Pt electrode is set parallel to the 71° domain walls and grounded. A linearly polarized blue laser with a wavelength of 405 nm (*hv* = 3.06 eV) was used to illuminate the area between the Pt electrode and the tip-surface contact. Given the nanoscale tip-sample contact diameter (*r*_*tip*_ ~ 30 nm), measurement of the current by PhAFM tip could provide an insight into local photoelectric properties with same resolution as the contact diameter. Figure 2b shows the time evolution of local PV current (*I*_*PV*_) by switching on/off the laser. A sizable current is recorded by the conductive AFM tip without applying external voltage. The magnitude of *I*_*PV*_ could be easily tailored by tuning illumination intensity, as shown in Supplementary Figure S4. Although the tip-surface contact area is about five order of magnitude smaller than that of the in-plane electrodes, the local *I*_*PV*_ can reach the same order of magnitude as compared to the *I*_*SC*_ (330 pA) measured with in-plane electrodes (Fig. 1c) under the same illumination conditions. Meanwhile, the local *V*_*OC*_ can reach as large as -35 V determined by the extrapolation of the *I-V* characteristic in Fig. 2c (blue curve), which is one order of magnitude higher than the bandgap of BFO. Similar to the tip-enhanced photovoltaic effect in BFO single crystals, the external quantum efficiency of this ferroelectric solar cell is dramatically enhanced by at least five order of magnitude with the nanoscale contact^19^.

The first issue to solve is the origin of the PV current collected by the tip, respectively weather it is generated by a potential Schottky contact existing at the tip-BFO interface or by the BPV effect, as in the bulk case. For this we measured the PV current collected by the tip as function of the incident light polarization. A two-fold azimuthal dependence of *I*_*PV*_ as a function of the light polarization (Fig. 2d) reveals a similar behaviour as in the case of the macroscopic PV current (Fig. 1d), confirming that the driving force behind the tip-enhanced photovoltaic effect is also the BPV effect.

**Figure 3:**
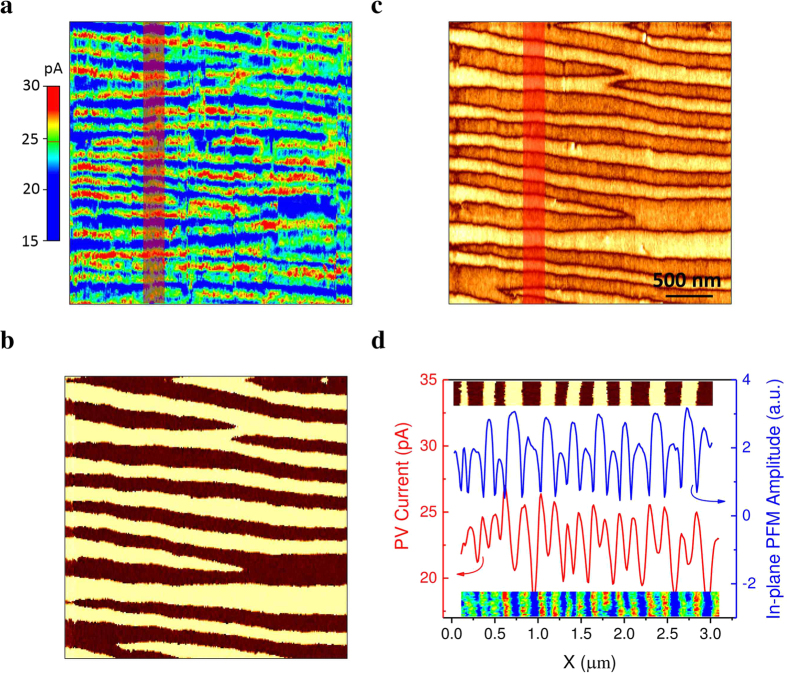
Spatially resolved photovoltaic current mapping with domain walls parallel to grounded electrode. (**a**) Spatial distribution of the photovoltaic current detected by PhAFM. (**b)** and (**c**) are the in-plane PFM amplitude and phase signal, respectively. (**d**) Profile analysis of photovoltaic current distribution and in-plane PFM amplitude signal averaged over the area marked by red in (**a**) and (**b**), upper insert and lower insert show the corresponding PFM phase images and the current distribution of the analyzed region, respectively.

Further on we proceed in acquiring a spatially resolved PV current distribution by scanning a conductive tip on the illuminated surface of BFO thin film and simultaneously recording the *I*_*PV*_. With the help of a switching system, the AFM can be operated sequentially in PhAFM mode and PFM mode, enabling precise correlation between the domain and PV current patterns in the same area. Figure 3a shows such correlation between *I*_*PV*_ and ferroelectric domains in a 3 × 3 μm^2^ area. We specially used a low laser intensity to set the average *I*_*PV*_ to a relative low value, leading to a high resolution of spatial current distribution. PV current map shows some intriguing features. Firstly, the PV current is detected over the whole scanned surface containing both domains and DWs, with an averaged value of 25 pA under this specific mapping condition. Secondly, and more important, the PV current was significantly enhanced at particular positions as highlighted by the bright lines in Fig. 3a. By comparing to the domain patterns revealed by both PFM in-plane phase signal (Fig. 3b) and in-plane amplitude signal (Fig. 3c), the lines exhibiting a higher PV current in Fig. 3a correspond to the locations where PFM amplitude signal is almost zero, respectively at the DWs. This correlation could be further validated by analysing the profiles of spatially distributed PV current and the in-plane PFM amplitude of the same region marked by red in Fig. 3a and b. As illustrated in Fig. 3d, each peak of PFM in-plane amplitude corresponds to a minimum of PV current while every minimum of the PFM in-plane amplitude, which is associated with the domain walls, points to a maximum of PV current. This clearly show a significant enhancement of local PV current at DWs compared to the bulk of the domain.

**Figure 4:**
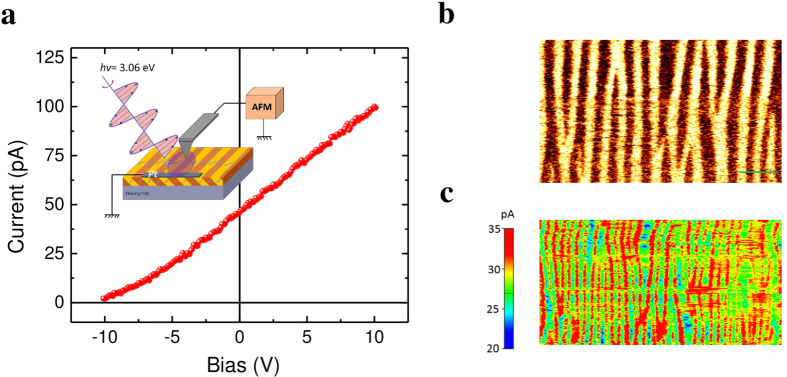
Spatially resolved photovoltaic current mapping with domain walls perpendicular to grounded electrode. (**a)** Local *I-V* curve acquired by PhAFM. The insert shows the measurement geometry schematically. (**b)** and (**c)** In-plane PFM phase signal and spatially-resolved photovoltaic current distribution of the same area, respectively. The length of the scale bar is 500 nm.

The same analysis can be performed with the grounded Pt counter electrode running perpendicular to the domain walls as illustrated in the insert of Fig. 4a. Figure 4a shows typical *I-V* characteristics measured in this perpendicular geometry, indicating a sizable PV current (46 pA) and above bandgap *V*_*OC*_ (~10 V), similar to the macroscopic case with in-plane perpendicular geometry (Fig. 1c). Following the same procedure, we map the *I*_*PV*_ distribution in this second case when the domain walls are running perpendicular to the Pt electrode. Likewise, the PV current is probed all over the scanned area with a significant enhancement at the DWs (see Fig. 4b,c).

### Nanoscale characterization of photoconductive current

**Figure 5:**
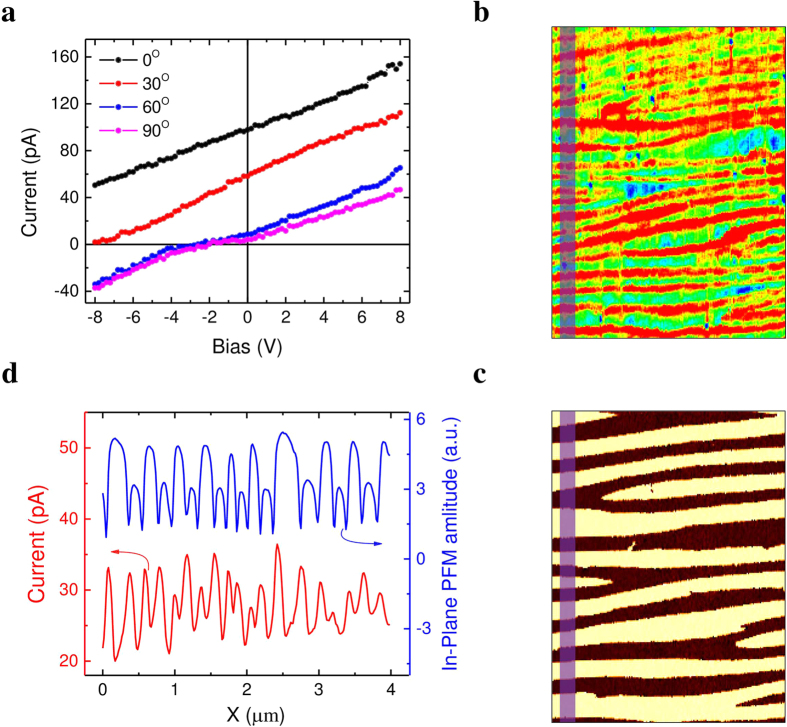
Spatially resolved photoconductive current mapping. (**a**) *I-V* characteristics under different incident light polarization angles. (**b)** Spatially resolved distribution of photoconductive current under an external bias of 5 V and (**c)** the PFM in-plane phase signal of the same area. (**d)** Profile analysis of in-plane PFM amplitude and the current distribution of the same region marked by red in (**b**) and (**c**). Here the grounded Pt electrode is parallel to the domain walls.

In order to understand our experimental results, we analyse the origins of the current measured through the tip. As been shown above that the main origin is the BPV effect, we may now consider the most general case of a uniformly illuminated ferroelectric film under an electric field. The current in a certain crystallographic direction *J*_*i*_ consists of three contributions, namely bulk photovoltaic effect, drift and diffusion as expressed below^20^


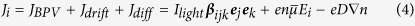


where *e* is the electron charge, *n* the carrier density, 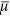 the effective mobility, *E*_*i*_ the electric field component along the specified direction and *D* the diffusion coefficient. The simple diffusion currents as well as the Dember effect resulting from non-uniform ill umination are neglected here due to uniform illumination in the area of interest. Keeping in perspective the light polarization dependence of BPV effect (see Fig. 2d), one can recognize the possibility to decrease the bulk photovoltaic current *J*_*BPV*_ by tuning the light polarization and map in such way only the distribution of drift current, i.e. photoconductive current. The latter according to Equation (4) depends solely on local carrier density *n* and effective mobility 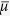. To verify this hypothesis, the *I-V* characteristics at different light polarizations are measured and shown in Fig. 5a. It is clear that *I-V* curves shift downwards along ordinate with increasing laser polarization angle from 0° to 90° and the PV current is largely suppressed when laser polarization parallel to the stripe domain walls (*θ* = 90°), which is consistent with light polarization dependence of PV current (see Fig. 2d). By applying 5 V to the conductive tip and setting the incident laser polarization angle as 90° (see Fig. 5b), only the photoconductive current is acquired through the AFM tip. The domain configuration of the same area is characterized by the in-plane PFM phase signal as shown in Fig. 5c. While certain photoconductive current is detected over the whole scanned area, a significant enhancement is observed at the DWs, as demonstrated by the profile analysis in Fig. 5d. This enhancement of the photoconductive current collected by the moving tip exactly at the domain walls is a consequence of a higher photoconductivity within the domain wall, which is consistent with in-plane macroscopic measurements showing enhanced photoconductivity when domain walls are aligned perpendicular to electrodes^13^. Although being a local property, this abnormal photovoltaic effect and enhanced photoconductivity of the domain walls is detectable only if the illumination is global. As shown in the Supplementary Fig. S5, illuminating only the area under the AFM tip fails to generate significant photovoltaic current. The whole area between the grounded electrode and the AFM tip needs to be illuminated in order to observe the effects.

## Discussion

In a simple scenario the domain walls showing a higher photoconductivity will play the role of a high conducting path for the photo-generated non-equilibrium carriers. If the illumination is local, only the local photoconductive properties are measured with external applied bias whereas illumination of the whole area between the collecting tip and counter electrode will add the photovoltaic current, which is a bulk property. We conclude from here that the local photovoltaic effect at the DWs is missing or negligible compared to the bulk PV effect. A higher photoconductivity is generally due to a higher carrier density *n* and/or effective mobility 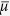. An enhanced carrier density of DWs is related to a different electronic structure within DWs, i.e. either higher defect density or Fermi level position or a combination which would result in a different transport process at DWs compared to the bulk^1^,^2^,^21^,^22^,^23^.

A higher carrier density at the domain walls would actually have a twofold effect on the local photocurrent enhancement. The first one is very obvious as explained above by enhancing the internal conductivity, and a second is to lower the effective tip-surface contact resistance. Apart from the contact region, the bulk of BFO film underneath the contact also plays an important role in the transport process, especially considering the extremely high current density (~10 A/cm^2^) flowing through the tip-surface contact. *I-V* characteristics (Supplementary Fig. S5 and Fig. S6) acquired under illumination indicate an ohmic behaviour in the low voltage range (either external applied voltage or photo-emf arising from the BPV effect). Considering this nanoscale contact (*r*_C_ ~ 30 nm) as an ideal point contact geometry, the dependence of current on voltage can be expressed as^24^:


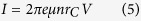


Accordingly, the transport resistance in the BFO film decrease as carrier density and/or mobility increases. Hence, domain walls, which possess higher carrier density and/or mobility, would possess higher conduction as compared to that of the domains. Consequently, in sharp contrast to the adverse effect upon *V*_*OC*_, domain walls could effectively facilitate the transport and collection of photovoltaic current generated in the bulk of ferroelectrics.

In summary, the local photovoltaic effect of BFO film consisting of pure 71° domain has been explored using the photo-AFM with a nanoscale resolution. PV current, shown to originate from the bulk photovoltaic effect, is detected over the entire surface and significant enhancement occurs at the domain walls. Moreover, by using the ability to tune the BPV effect by light polarization, it is established that the enhancement of PV current at the domain walls is due to their higher photoconductivity and not a photovoltaic effect within domain walls.

## Methods

### BFO film growth

Epitaxial BFO films were fabricated by pulsed laser deposition (PLD) technique on TbScO_3_ (110) substrates at 670 °C in 0.15 mbar oxygen pressure. The laser energy density was 1 J/cm^2^ and the laser repetition rate was kept at 10 Hz. After deposition, the films were cooled down at 15 °C/min and in 200 mbar oxygen pressure. In order to get pure 71° domain walls, TbScO_3_ substrates were annealed at 1000 °C with O_2_ flow of ~200 sccm for 2 hours before film deposition^11^.

### APV effect measurement

Anomalous photovoltaic effect was first characterized by in-plane platinum electrodes with a defined gap of about 80 μm and a length of about 300 μm. BFO surface was illuminated by a 405 nm laser (Newport LQA405-85E) with an intensity of 55 W/cm^2^. The incident laser polarization was tuned by rotating half wavelength plate (Thorlabs).

### Photoelectric atomic force microscopy

Local electronic properties were characterized and mapped using a home-made PhAFM system based on an AFM system (XE-100, Park), as depicted in Figure S3 of SI. Here, the distance between the tip contact and grounded Pt electrode is about 40 μm.

### Piezoresponse force microscopy

PFM was performed with ac voltage of 2.5 V amplitude and 19.27 kHz frequency applied on AFM tip (HQ: NSC14/Pt, MikroMasch).

## Additional Information

**How to cite this article**: Yang, M.-M. *et al*. Enhancement of Local Photovoltaic Current at Ferroelectric Domain Walls in BiFeO_3_. *Sci. Rep.* **7**, 43070; doi: 10.1038/srep43070 (2017).

**Publisher's note:** Springer Nature remains neutral with regard to jurisdictional claims in published maps and institutional affiliations.

## Supplementary Material

Supplementary Information
