# Supplementary material for: Enhancement of Local Photovoltaic Current at Ferroelectric Domain Walls in BiFeO3
Source: Sci Rep. 2017 Feb 20;7:43070. doi: 10.1038/srep43070 (PMC5380211; doi:10.1038/srep43070)
Supplement: Supplementary Information [file srep43070-s1.pdf]

Supporting Information

Enhancement of Local Photovoltaic Current at Ferroelectric Domain walls in $BiFeO_3$

Ming-Min Yang, Akash Bhatnagar, Zheng-Dong Luo, Marin Alexe**

Ming-Min Yang, Dr. Akash Bhatnagar, Zheng-Dong Luo and Prof. Marin Alexe
University of Warwick, Coventry, CV4 7AL, United Kingdom
E-mail: Mingmin.Yang@warwick.ac.uk; M.Alexe@warwick.ac.uk

Dr. Akash Bhatnagar
Centre for Innovation Competence SiLi-Nano, Karl-Freiherr-von-Fritsch-Straße 3, D-06120 Halle (Saale), Germany.

BFO Film Structure and Quality

As shown in **Figure S1a**, the XRD 2θ-ω scan of BFO film grown on $TbScO_3$(110) substrate (TSO) only shows strong (*00l*) (*l*=1,2) peaks, indicating pure phase of BFO film. Due to the small lattice mismatch between BFO and TSO, the diffraction peaks of BFO and TSO almost coincide with each other. The reciprocal space maps (RSM) near $\{002\}_{pc}$ faces at both orthogonal in-plane sample orientation, i.e., $\varphi=0^{O}$ and $\varphi=90^{O}$, show one peak of BFO film without peak splitting, confirming the non-tilt nature of lattice arrangement of BFO film on TSO substrate (see **Figure S1c** and **d**). Splitting of BFO $\{113\}_{pc}$ faces shown in **Figure S1d** and **f** indicates two ferroelastic variants, namely r_1 and r_4, exist in BFO film, as depicted in **Figure S1b**. Therefore, these XRD measurements prove only two ferroelastic variants (r_1^+ and r_4^+) coexist in BFO film resulting in 71° domain walls.

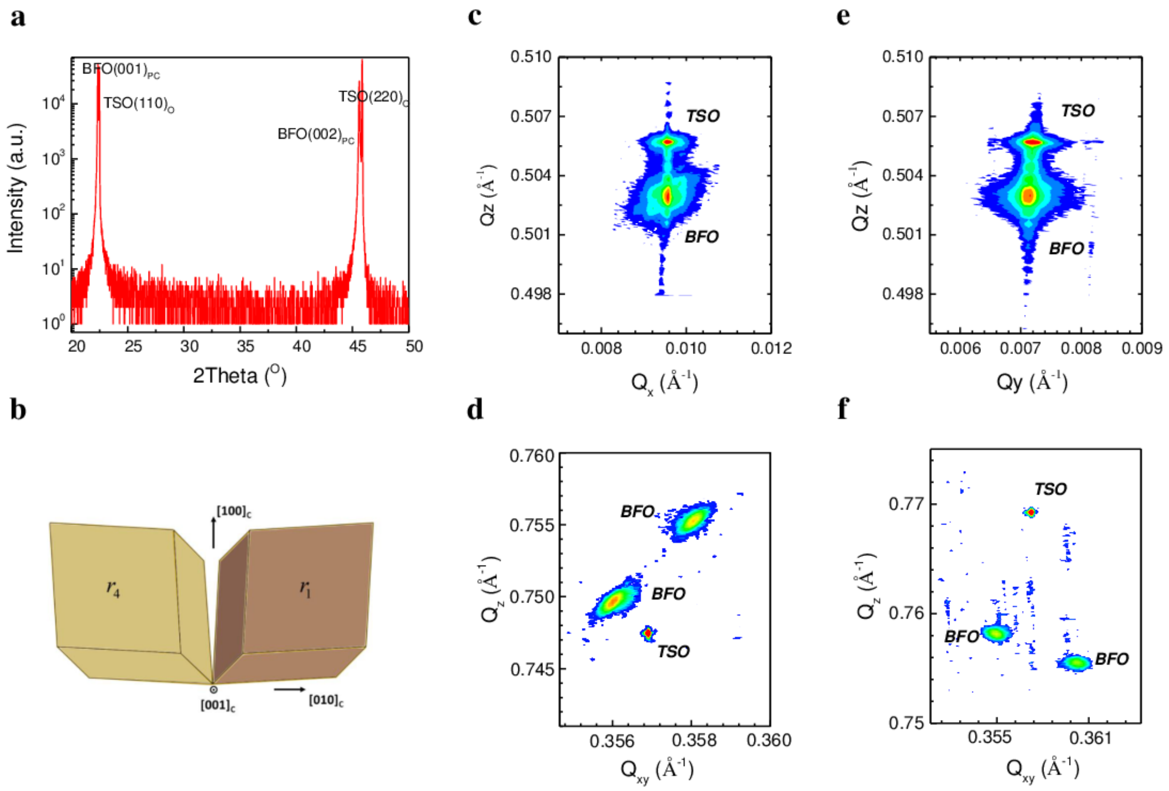

Figure S1. XRD characterization of $BiFeO_3/TbScO_3$ film. **a)** X-ray 2θ-ω scan of pseudocubic (002) face. **b)** Schematic of lattice structure. Reciprocal space maps **c)** and **d)** are the pseudocubic {002} face at φ of 0° and 90°, respectively. **e)** and **f)** are the reciprocal space maps of pseudocubic {113} measured at φ of 45° and 135°, respectively.

Illumination geometry for measuring the bulk photovoltaic effect

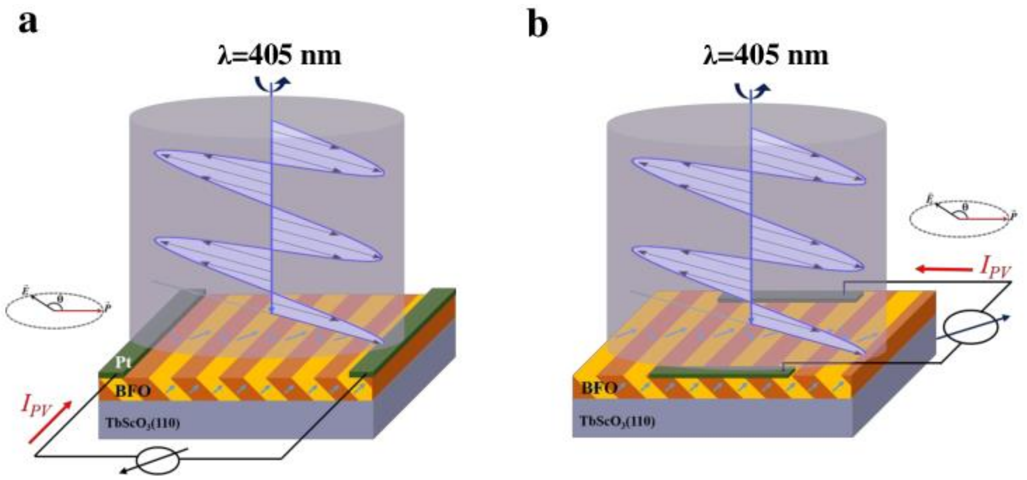

Figure S2. In-plane electrode geometry and illumination geometry for macroscopic measurements. Electrodes aligned **a)** parallel to and **b)** perpendicular to 71° domain walls.

Photovoltaic tensor and its values for BFO

Table S1. Conversion law between tensor notation and matrix notation [1]

Tensor notation	11	22	33	23, 32	31, 13	12, 21
Matrix notation	1	2	3	4	5	6

Then the transformation between tensor notation and matrix notion can be expressed below for BPV tensor elements:

$$\beta_{15} = \beta_{24} = 2\beta_{113} = 2\beta_{131} = 2\beta_{223} = 2\beta_{232};$$

$$\beta_{22} = -\beta_{21} = -\beta_{16} = \beta_{222} = -\beta_{211} = -2\beta_{112} = -2\beta_{121}; \quad (1)$$

$$\beta_{31} = \beta_{32} = \beta_{322} = \beta_{311};$$

$$\beta_{33} = \beta_{333}.$$

Table S2. Values of the BPV tensor elements of BFO film

BPV tensor elements	Value (V^{-1})
β_{15}	-6.5×10^{-5}
β_{31}	1.81×10^{-4}
β_{33}	2.11×10^{-4}
β_{22} [2]	1.10×10^{-4}

The experimental results shows excellent agreement with the PV current predicted by BPV model as expressed by **Equation 2** and **3** in the main text (see Figure 1d). Using the reported value of β_{22} [2], the values of rest three independent BPV tensor elements can be calculated which is shown in **Table S2**.

Photoelectric AFM setup

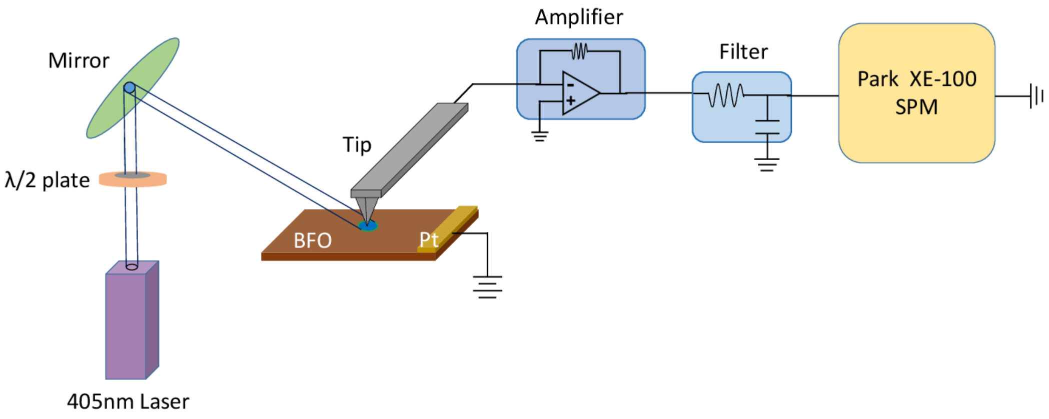

Figure S3. **Schematic for photoelectric atomic force microscopy (PhAFM).** $BiFeO_3$ film is illuminated by 405 nm laser with tunable light polarization realized by rotating half wavelength (λ/2) plate. The currents detected by the conductive tip are first read by a trans-impedance amplifer with a gain of 10^8 V/A. The output voltage signal is further amplified by a factor of ten and processed by a low band pass filter. Lastly, the processed voltage signal was the fed back to the Park XE-100 SPM system in order to simultaneously record the topography and the photoelectric current.

Dependence of PV current of the light intensity

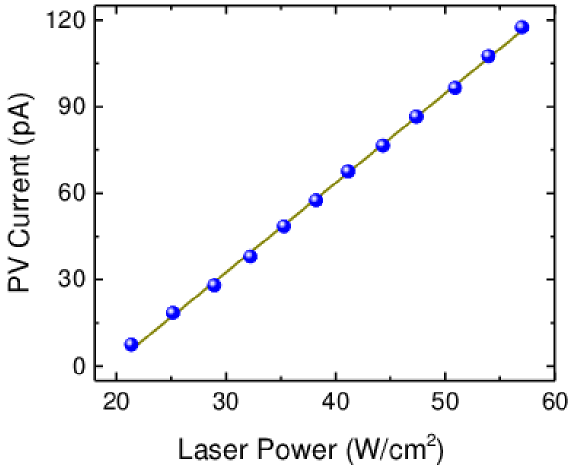

Figure S4. Photovoltaic current collected by a Pt-coated AFM tip in contact with BFO film as a function of illumination intensity. The yellow line is a linear fit of the experimental data shown as blue dots.

Transport mechanism in tip-enhanced photovoltaic effect.

Understanding the transport mechanism of tip-enhanced PV effect would reveal the mechanism behind the enhanced PV effect at domain walls. As demonstrated by Figure 2d, the BPV effect is the driving force of the local PV current probed by the AFM tip. According to the theory of the BPV effect, photo excitation gives rise to asymmetric momentum distribution of photogenerated carriers in *k*-space, resulting in either a PV current flowing towards particular crystallographic directions in the short-circuit condition or a large photo-emf under open-circuit condition[3,4]. In the PhAFM measurement geometry (see Figure 2a), illumination of the BFO film with above bandgap light would lead to a potential difference between the tip and the BFO surface in open-circuit condition and a continuous PV current when the tip is contact with the BFO surface. Note here it is not only the BFO surface underneath the tip but all the illuminated area between the tip and the grounded Pt electrode that provide the driving force for the PV current in the tip-enhanced PV effect. Hence, the illuminated BFO surface between the tip contact and the Pt electrode plays both roles, respectively conduction path and power source, as depicted in Figure S6a. Supporting evidences for this scenario are provided by a two-laser experiments as shown in the Figure S6b. A laser, labelled as A, illuminating only the area near the tip contact induces a negligible PV effect, while simultaneously illuminating the surface close to the Pt electrodes by another laser, labelled as B in Figure S6c and d, gives rise to a substantial PV effect. From this simple experiment it is clear that a large PV current appears only when the whole area between the Pt electrode and tip is illuminated. This clearly confirms the role of illuminated BFO surface as driving source for tip-enhanced photovoltaic current.

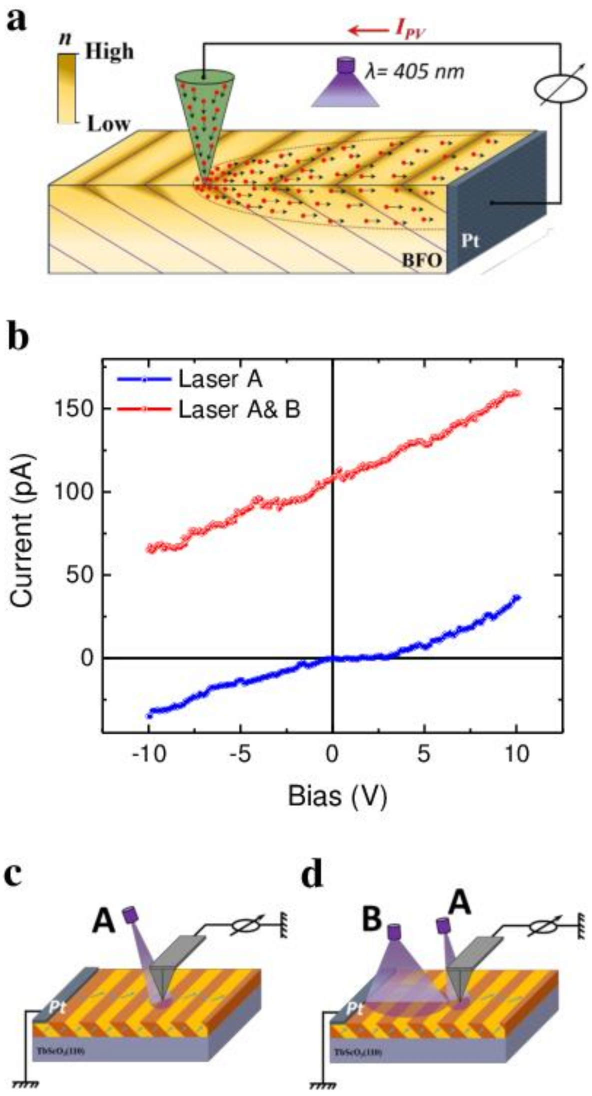

Figure S5. Origin of the tip-enhanced photovoltaic effect. a) A schematic showing the electronic transport process in the tip-enhanced PV effect. Illumination upon BFO surface with light wavelength of 405 nm excites nonequilibrium carriers over the whole illuminated area while enhanced carrier density n exsits near domain walls, as illustrated by the dark yellow. Due to the BPV effect, part of the excited carriers possess directional momentum resulting in PV current flowing along the direction of the in-plane ferroelectric polarization, which is labled as red dots with arrows. **b)** *I-V* curves obtained in two laser experiment. Blue *I-V* curve is obtained with only Laser A illuminating the tip-contact region as schematically shown in **c)** while red *I-V* curve is the one measured with illimination from both A and B as depicted in **d)**.

Tip-surface electric contact

Apart from the driving force, the local conduction also plays an important role in the local PV effect. The conduction path of the photo-generated carriers consists of tip-surface junction, bulk of BFO film and BFO/Pt interface. The tip-surface contact, which in general case is a Schottky contact, would play the limiting role in the current transport due to its much higher contact impedance than that of the bulk, especially in the reverse direction[5-7]. However, under illumination with above bandgap energy photon the quasi-Fermi level rise significantly, lowering and thinning the barrier, even froming ‘Ohmic contact’[8,9]. Consequently, the effective contact impedance would be largely reduced under illumination and conductive tip can efficiently collect non-equilibrium carriers resulting in a tip-enhanced photovoltaic effect. In addition, the higher carrier density at domain walls, as demonstrated by spatially resolved photoconductive current mapping, could further reduce the local contact resistance at domain walls.

Ohmic behavior of the tip-BFO contact under illumination

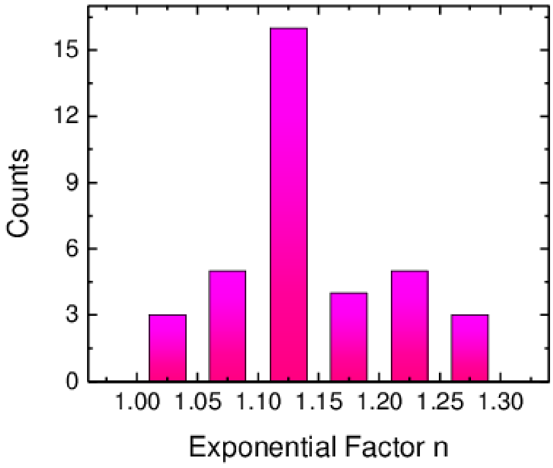

Figure S6. Distribution of exponential factor *n* of the current-voltage characteristics considering a power law $I \propto V^n$ dependence. The *I-V* characteristics were acquired through the Pt-coated AFM tip in different places under different illumination conditions.

References

1 Nye,J. F., *Physical Properties of Crystals*, (Oxford University Press, Amen House, London, 1957).

2 Ji, W., Yao, K. and Liang, Y. C., Evidence of bulk photovoltaic effect and large tensor coefficient in ferroelectric $BiFeO_3$ thin films*Physical Review B* **2011**, 84, 094115.

3 Fridkin, V. M., Bulk photovoltaic effect in noncentrosymmetric crystals, *Crystallography Reports* **46,** 654-658(2001).

4 Yang,M., Bhatnagar A., and Alexe, M., Electronic Origin and Tailoring of Photovoltaic Effect in $BiFeO_3$ Single Crystals. *Adv. Electron. Mater.* **1,** 1500139(2015).

5 Seidel, J. *et al.*, Domain wall conductivity in La-doped $BiFeO_3$.*Phys. Rev. Lett.* **105,** 197603(2010).

6 Maksymovych, P., Jesse,S., Yu, P., Ramesh, R., Baddorf, A. P., Kalinin,S. V., Polarization control of electron tunneling into ferroelectric surfaces. *Science* **324**, 1421(2009).

7 Simmons, J. G., Transition from electrode-limited to bulk-limited conduction processes in metal-insulator-metal systems. *Phy. Rev.* **166,** 912(1968).

8 Schwartz, B., *Ohmic contacts to semiconductors*. (Electronics Division, Electrochemical Society, New York, 1969).

9 Rideout, V. A review of the theory and technology for ohmic contacts to group III–V compound semiconductors *Solid-State Electron*. **18,** 541(1975).
